# Scleral buckling under a slit-lamp illumination system in patients with primary rhegmatogenous retinal detachment

**DOI:** 10.1186/s40942-026-00913-3

**Published:** 2026-08-06

**Authors:** Vasileia Chatzistergiou, Boris Marguier, Pierre Negrier, Aude Couturier, Elise Philippakis

**Affiliations:** 1 Ophthalmology Department, Université Paris Cité, AP-HP, Hôpital Lariboisière, 2 Rue Ambroise Paré, Paris, 75010 France; 2 Ophthalmology Department, Hôpital Fondation Adolphe de Rothschild, 29 Rue Manin, Paris, 75019 France

**Keywords:** Rhegmatogenous retinal detachment, Retinal detachment, Scleral buckling, Slit-lamp, Surgery, Vitreoretinal surgery

## Abstract

**Purpose:**

To present the slit-lamp illumination and visualisation system for scleral buckling for primary rhegmatogenous retinal detachment (RRD) and assess its feasibility and postoperative outcomes.

**Methods:**

In a retrospective, single-center, non-comparative case series, patients with subtotal RRD who underwent scleral buckling under a slit-lamp illumination system at Lariboisière Hospital, between May 2017 and May 2022, were included. Patients who underwent scleral buckling combined with vitrectomy, with a history of retinal surgery and with maculopathies interfering with visual acuity were excluded. The following preoperative data were collected: best-corrected visual acuity (BCVA), number and location of retinal breaks, extend of the detachment, presence of macular detachment. The fundus was visualised using a quadraspheric lens, and the slit-lamp system integrated in the surgical microscope. Anatomical success rate, BCVA improvement and complications were collected.

**Results:**

Forty-six eyes of 46 patients with a mean age of 43.2 ± 12.8 years, followed for 8.7 ± 8.3 months were included. No procedure was switched to vitrectomy during surgery. The single surgery success rate was 93.5% (43 eyes). Three eyes had to undergo vitrectomy due to vitreoretinal proliferation. BCVA improved from 0.63 ± 0.76 LogMAR (Snellen 20/80) before surgery to 0.33 ± 0.40 LogMAR (Snellen 20/40) after surgery (p=0.002). Intraoperative and postoperative complications were experienced by two and nine patients, respectively.

**Conclusion:**

Scleral buckling using a surgical microscope with an integrated slit-lamp and a wide-angle contact lens is a feasible technique associated with satisfactory anatomical and functional outcomes. This approach was associated with a high single-surgery success rate and a manageable complication rate.

**Clinical trial number:**

Not applicable.

**Supplementary Information:**

The online version contains supplementary material available at https://doi.org/10.1186/s40942-026-00913-3.

## Introduction

Primary rhegmatogenous retinal detachment (RRD) is due to changes in vitreoretinal adhesions and tractional forces that promote retinal tears, and to the displacement of the liquefied vitreous into the subretinal space. Subretinal fluid drainage and subsequent treatment of the retinal tear are essential to effectively manage RRD [1–3]. Scleral buckling (SB) is an effective surgical technique that has remained almost unchanged over the years [4–7]. The principle is to provide external indentation at the retinal tear site, creating an adhesion between the sensory retina and the retinal pigment epithelium and modifying the intraocular fluid movement, which pushes the detached retina back into its original position. In large cohorts, the success rate of SB is 91.2% [8]. Identifying all retinal breaks and treating them with cryopexy is essential to the success of SB, and clear visualisation of the fundus is thus mandatory at all surgical steps. To date, indirect ophthalmoscopy is the reference technique for visualising the retina during surgery [9]. Over time and with the development of intraoperative microscopes, other visualisation modalities have been used, such as the 25-gauge chandelier, and a slit-lamp illumination system integrated into the intraoperative microscope. Although the feasibility of the integrated slit-lamp illumination system has been previously described, the technique is not yet widely used [10]. The aim of this study was to present the integrated slit-lamp visualisation technique for SB, and to assess the feasibility and the anatomical and visual outcomes of the technique. The primary outcome measure was the proportion of eyes achieving successful retinal reattachment after a single surgery. The secondary outcome measures were the gain in visual acuity and the occurrence of complications.

## Materials and methods

Patients with primary RRD who underwent SB under a slit-lamp illumination system with a wide-angle-viewing contact lens at Lariboisière Hospital between May 2017 and May 2022 were retrospectively included in this study. Of note, slit-lamp visualisation is the only technique used for SB in the department. Informed consent to participate in the study and to publish the study was obtained from all patients. The study adhered to the tenets of the Declaration of Helsinki and its conduct received a favourable opinion from the Ethics Committee of the French Society of Ophthalmology (IRB 00008855 Société Française d’Ophtalmologie IRB#1). No funding was received for conducting this study. Patients who underwent SB combined with pars plana vitrectomy, with a history of retinal surgery and with maculopathies that could interfere with the visual acuity (macular oedema, age-related macular degeneration, macular hole, etc.) were excluded.

The following preoperative data were collected from each patient: age, gender, lens status, best-corrected visual acuity (BCVA), intraocular pressure, presence of a single or multiple retinal breaks, location of retinal breaks, extend of the peripheral detachment measured in clock hours, presence of a macular detachment. Ultra-wide-field fundus photography (Optos® California), and macular spectral-domain optical coherence tomography (SD-OCT: Spectralis® OCT, Heidelberg Engineering, Germany) scans were also acquired. Intraoperative data, including external subretinal fluid drainage and complications, were also collected. Finally, postoperative data, including the achievement of anatomical success after primary surgery, immediate and late complications, the BCVA between three and six months postoperatively and the need for additional procedures, were collected.

Data were analysed using EasyMedStat v3.29. Continuous variables are presented as a mean ± standard deviation (SD) (range) and categorical variables as a percentage (%). The Shapiro-Wilk test was used to assess data normality. A paired-sample t-test was used to compare preoperative and postoperative data. For the statistical analysis, the BCVA was converted into logarithm of the minimum angle of resolution (LogMAR). For all tests, a p value < 0.05 was considered significant.

### Surgical procedure

All surgical procedures were performed using a surgical microscope (Zeiss Lumera® 700, Carl Zeiss meditec AG, Jena, Germany). All procedures were performed by five different surgeons with years of experience in vitreoretinal surgery. Extraocular muscles, except for the two oblique muscles, were isolated at their insertion, and 4/0 silk traction sutures were placed. The fundus was visualised using a wide-angle-viewing quadraspheric contact lens (Superquad 160; Volk®, Volk Optical, Inc., US), and the slit-lamp illumination system integrated in the surgical microscope. The slit lamp could be set in two different positions (45° to the right or left) and could be moved horizontally and vertically. The illumination system had an amplitude extending from the optic nerve to the ciliary bodies by tilting the eye or indenting its surface, if needed. A scleral indenter was used to indent and identify retinal tears, and transscleral cryotherapy was applied on them. Still using the microscope, a silicone sponge was sutured to the sclera using a double-crimped 5/0 Mersuture mattress suture. In the presence of a significant amount of fluid in the retinal break area, subretinal fluid was drained by transscleral puncture. Otherwise, an anterior chamber puncture was performed to achieve transient hypotony prior to tightening of buckling stitches. The appropriate position of SB was then checked. (Supplementary Material 1)

## Results

### Preoperative characteristics

Sixty-four patients had undergone SB alone. Fourteen patients who had previously undergone retinal surgery and four with a maculopathy were excluded. Finally, 46 eyes of 46 patients (22 women and 24 men) with a mean age of 43.2 ± 12.8 years (range: 19–81) were included. The mean preoperative BCVA was 0.63 ± 0.76 LogMAR (Snellen: 20/80 ± 20/180). The mean follow-up duration was 8.7 ± 8.3 months (range: 1–32). All patients except one were phakic. The macula was detached in 21 patients (45.6%).

Seventeen patients (37%) had a single retinal break, 26 (57%) had multiple retinal breaks, and three (6%) had a retinal break which had not been identified during the preoperative examination while it was discovered intraoperatively. The two most common retinal break locations were in the inferior quadrant for 21 eyes (46%) and in the superior quadrant for 17 eyes (37%). The mean extend of retinal detachment was 4 ± 1.7 clock hours (range: 1–10). The two most common retinal detachment locations were in the inferior quadrant for 23 eyes (50%) and in the temporal quadrant for 15 eyes (33%). Demographics and preoperative parameters are presented in Table 1.

**Table 1:** Preoperative characteristics of the population of patients with retinal detachment operated with scleral buckling under a slit-lamp illumination system

Characteristics	
Eyes (number)	46
Sex (male/female)	24 / 22
Age (years)	43.2 ± 12.8
Follow-up (months)	8.7 ± 8.3
Best-corrected visual acuity (LogMAR)	0.63 ± 0.76
Retinal breaks (number)	1.83 ± 0.9
Macular involvement (number)	21
Extend of retinal detachment (clock hours)	4.09 ± 1.7
Phakic eyes	45

### Intraoperative findings and postoperative outcomes

No procedure was switched to vitrectomy. Subretinal fluid was drained in 18 patients (39%).

**Fig. 1:**
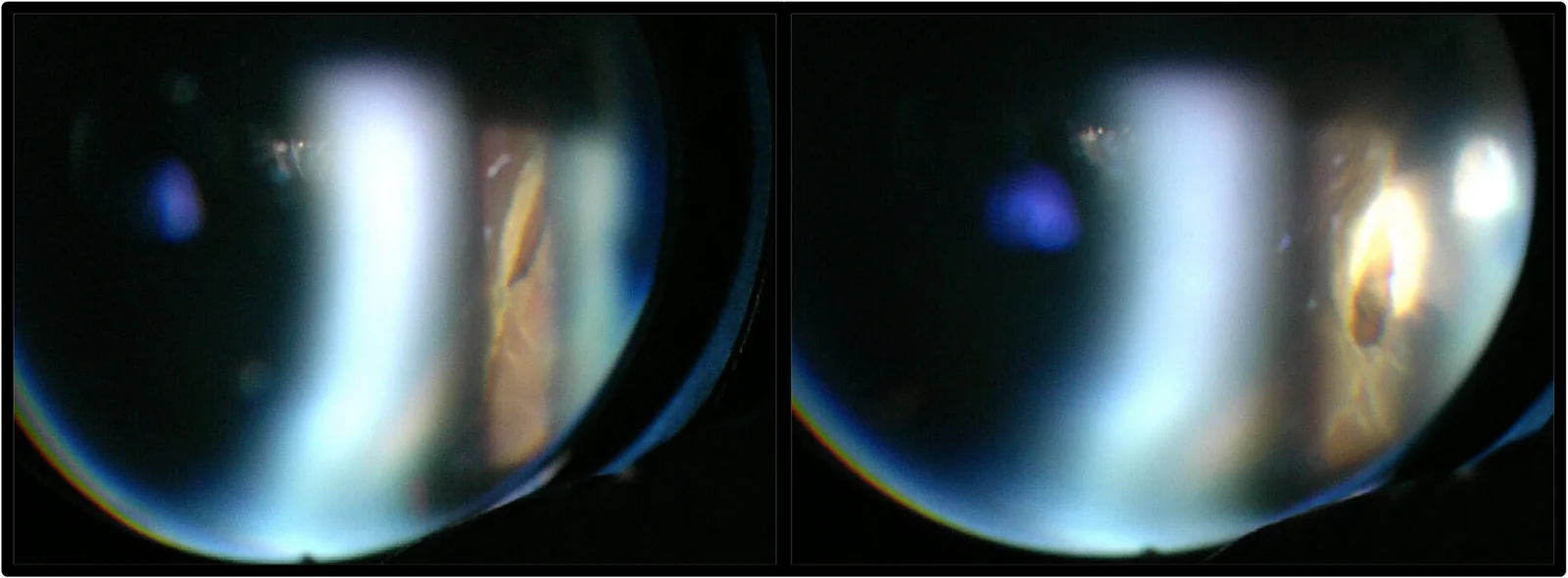
Intra-operative visualisation of a retinal break before (left) and after (right) cryoapplication using a wide-angle-viewing contact lens under a slit-lamp illumination system

**Fig. 2:**
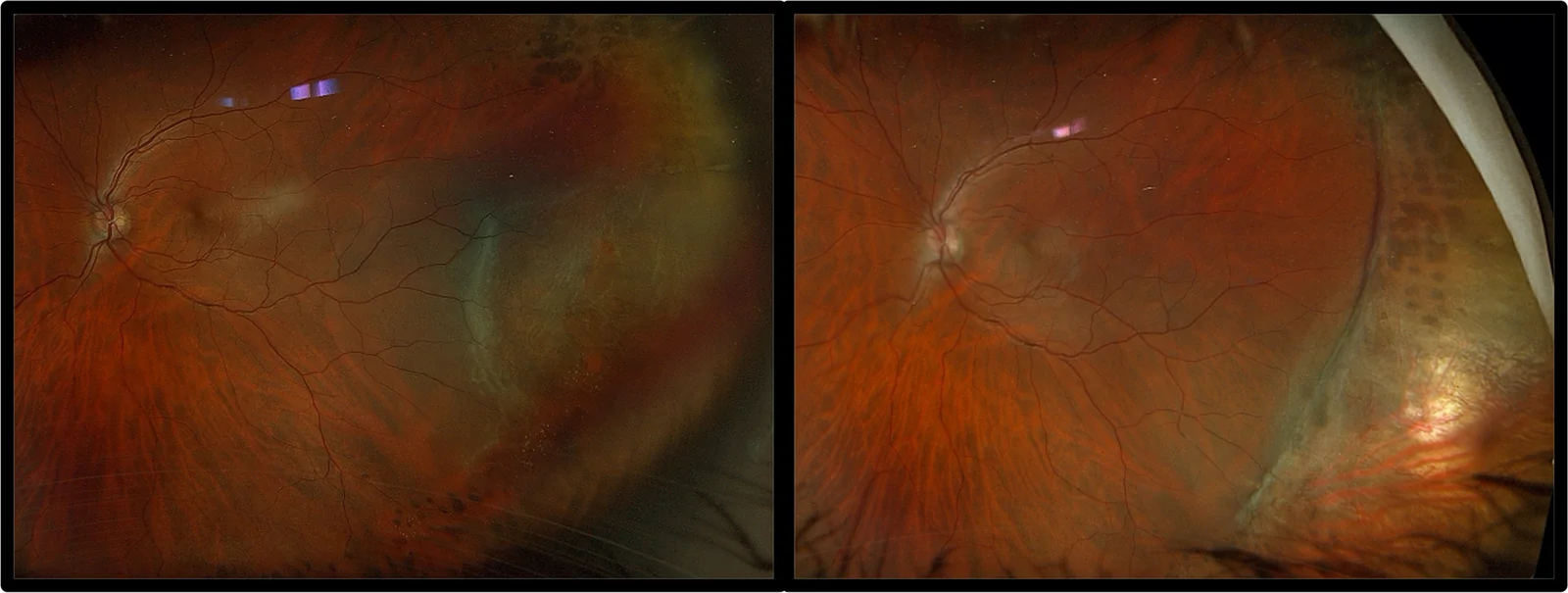
Retinal detachment before (left) and after (right) surgery with visible indentation on ultra-wide-field fundus photography

The single surgery success rate was 93.5% (43/46 eyes; 95% CI, 82.1–98.6%). (Figures 1 and 2) Three patients had to undergo a second surgery: two patients underwent vitrectomy, and the third one underwent SB re-fixation combined with vitrectomy, resulting in anatomical success. Among these three patients, none experienced any complications during vitrectomy. All vitrectomy procedures were performed for proliferative vitreoretinopathy. No retinal tears were missed during initial SB surgery.

The BCVA significantly improved from 0.63 ± 0.76 LogMAR (Snellen: 20/80 ± 20/180) before surgery to 0.33 ± 0.40 LogMAR (Snellen: 20/40 ± 20/50) after surgery (*p* = 0.002). A descriptive subgroup analysis according to macular status was also performed. Twenty-five eyes (54.4%) presented with macula-on retinal detachment and twenty-one eyes (45.6%) with macula-off retinal detachment. As expected, postoperative visual outcomes were generally better in the macula-on group. At postoperative month 1, mean BCVA was 0.08 ± 0.11 LogMAR in macula-on eyes and 0.48 ± 0.47 LogMAR in macula-off eyes. Given the limited sample size and the descriptive nature of this subgroup analysis, no formal statistical comparisons were performed.

Two patients (4.3%; 95% CI, 0.5–14.8%) experienced intraoperative complications: one had a subretinal haemorrhage during subretinal fluid drainage without macular involvement, and one had a haemorrhagic choroidal detachment secondary to ocular hypotension during subretinal fluid drainage. Nine patients (19.6%; 95% CI, 9.4–33.9%) experienced postoperative complications. Two (4.3%; 95% CI, 0.5–14.8%) had inflammatory macular oedema, two (4.3%; 95% CI, 0.5–14.8%) had a macular pucker, two (4.3%; 95% CI, 0.5–14.8%) had diplopia, two (4.3%; 95% CI, 0.5–14.8%) had intraocular hypertension, and one patient (2.2%; 95% CI, 0.1–11.5%) experienced sponge extrusion. Three patients (6.5%; 95% CI, 1.4–17.9%) required surgery: one for sponge removal and strabismus surgery, one for strabismus surgery, and one for sponge removal.

## Discussion

Before the advent of pars plana vitrectomy, SB was the only therapeutic option for retinal detachment. Given the progression and simplification of the vitrectomy procedure, SB may be considered a more complex procedure, especially for younger surgeons, requiring a longer learning curve, mainly due to the use of indirect ophthalmoscopy to visualise the retina. As a result, its use has declined, but it remains useful, especially in case of phakic retinal detachment or retinal detachment without vitreous detachment [11, 12]. In this study, SB was performed using a wide-field surgical microscope with the slit lamp integrated into the microscope technology used by all surgeons in our department as initially described by Bonnet, McPherson and more recently by Ohji [13–15]. 

Regarding the outcomes, a complete retinal reattachment after a single surgery was achieved in 93.5% of our 46 eyes with primary RRD that underwent SB using a wide-angle-viewing contact lens under a slit-lamp illumination system. This success rate, in a sample selected for SB, is in the range reported in prior SB series (93.33% and 95.63%) [16, 17]. Functional recovery was achieved with a significant improvement in visual acuity after surgery. This is consistent with the functional outcomes achieved after RRD surgery reported in the literature, regardless of the technique used (i.e., SB or pars plana vitrectomy) [18–20]. 

In our series, the intraoperative complications that occurred during SB were a subretinal haemorrhage and a choroidal detachment, due to subretinal fluid drainage and hypotony. Only one eye (2.2%) experienced a choroidal detachment. This percentage was lower than the 21.7% and 6.3% reported in previous studies [18, 21]. This could be explained by the fact that in these studies, subretinal fluid drainage was more frequently performed than in our series (100% versus 39%), resulting in a higher percentage of related complications.

Postoperative complications occurred in nine eyes (19.6%). Two patients experienced an intraocular pressure rise that was easily managed with topical treatment. The rate of postoperative macular oedema and macular pucker in our study was lower than that reported in previous studies using SB or vitrectomy in pseudophakic and aphakic eyes (macular pucker: 22.2% and 8.5% *versus* 4.3% in our study, macular oedema: 8.7% and 4.2% *versus* 4.3% in our study) [18, 21]. These higher complication rates could be due to the fact that pseudophakic or aphakic eyes were operated on for the second time, increasing the risk of inflammatory reactions [22]. Muscle dysfunction leading to diplopia was experienced by 4.3% of our patients, a rate similar to that reported in previous SB studies [18]. 

Integrated slit-lamp visualisation achieved satisfactory anatomical and functional outcomes in this series. Thus, other parameters such as the time, accessibility, ergonomics and cost-effectiveness may influence the surgeon’s choice of this technique. Regarding SB, the integrated slit-lamp visualisation system provides a more comfortable and ergonomic surgical setting than indirect ophthalmoscopy, with a sitting position and standard head posture. Many vitreoretinal surgeons suffer from neck and back injuries and using a surgical microscope instead of an indirect ophthalmoscope improves ergonomics [23, 24]. Treatment of retinal detachment with indirect ophthalmoscopy requires changing position during the same procedure, which is one of the aspects surgeons tend to avoid. Additionally, it allows teaching and recording, as the visualisation is shared within the microscope image, unlike indirect ophthalmoscopy, thus enhancing the training process for younger surgeons.

Regarding chandelier-assisted endo-illumination, ergonomics may be broadly similar, as both techniques allow a seated position with standard head posture. Regarding the retinal visualisation performance, the slit lamp is an additional light source integrated into the microscope that produces a bright light resulting in a well-defined slit image allowing observation of the anterior and posterior segments of the eye with high contrast. The orientation of the slit position (right, left) and the choice of the slit width (0.2 mm, 2 mm, 3 mm and 4 mm), combined with a wide-field contact lens, allow a magnified examination of the fundus under panoramic viewing conditions. Thus, it is easy to switch to a nasal or temporal examination, unlike the chandelier, which is most often inserted on the side opposite to the retinal tear. Thanks to dynamic scleral depression, the precise location of peripheral lesions can be determined. The quadraspheric lens also allows visualisation through air and gas bubbles. However, the microscopic examination of the retina offers a narrower angle than the broad enhanced panoramic surgical view obtained with chandelier illumination [25]. Compared with chandelier-assisted SB, slit-lamp illumination avoids scleral penetration and the use of disposable endoillumination devices, but provides a narrower panoramic view than modern chandelier-assisted wide-angle visualization systems. In our study, slit-lamp illumination combined with scleral indentation identified retinal tears that had not been detected on preoperative examination in three eyes, allowing all breaks to be treated during the same procedure. Visualisation of the peripheral retina with slit-lamp biomicroscopy is often more challenging in pseudophakic eyes. During SB, scleral indentation alleviates this issue. In our series, no difficulty in visualising the peripheral retina was encountered, either in phakic patients or in the only pseudophakic patient.

Moreover, chandelier-assisted endo-illumination requires the insertion of the fibre into the sclera, which can lead to complications: although rare, iatrogenic lens damage, liquefied vitreous leakage at the sclerotomy site of the chandelier insertion, conjunctival pigmentation at the chandelier illumination insertion site and endophthalmitis have been reported [26–29]. These specific complications related to sclerotomy have not been reported with slit-lamp visualisation, which does not require scleral penetration. Indeed, the risk of phototoxicity is higher with chandelier lighting as the light is not filtered by the lens, and the distance to the retina is shorter [30]. 

Nowadays, cost-effectiveness and ecological concerns should be considered since surgery is becoming less invasive thanks to more economical and greener solutions. Chandelier endo-illumination is a disposable instrument that also uses a vitrectomy generator, which may be of importance when resources are limited. In contrast, slit-lamp visualisation integrated into the microscope requires only a re-sterilisable quadraspheric lens.

Our study has some limitations, mainly due to its retrospective design and the inclusion of consecutive cases that underwent SB. The cases undergoing SB are already selected within the population of patients with retinal detachments as good candidates for surgical success, which may have contributed to the high single surgery success rate observed in our series and may limit the generalizability of the results. In routine clinical practice, subtotal retinal detachments are more frequently managed with SB, whereas more extensive or complex retinal detachments are often preferentially treated with pars plana vitrectomy. Accordingly, all patients included in our series had subtotal retinal detachments. However, the same bias applies to all recent studies assessing the outcomes of RRD surgery, since no strict guidelines have been defined regarding the best treatment option. Secondly, our study did not compare the outcomes of the different visualisation systems used for SB surgery, as only slit-lamp visualisation is used in our department, therefore, no conclusion regarding the superiority of this visualization technique over chandelier-assisted SB or other surgical approaches can be drawn. Nevertheless, none of the surgeons decided to switch to vitrectomy or to use a chandelier light during the procedure. Finally, the SB technique requires a learning curve, and the fact that multiple surgeons operated on the patients in our series could have led to a potential bias in decision making and complication rates. However, all surgeons involved were experienced vitreoretinal specialists with a comparable number of years of practice in vitreoretinal surgery. Although a learning curve remains necessary for peripheral retinal examination with slit-lamp illumination and scleral indentation, surgeons familiar with microscope-based vitreoretinal surgery may adapt rapidly due to the standard microscope ergonomics and shared surgical visualization.

In conclusion, in selected patients with RRD who are suitable candidates for SB, the use of a surgical microscope with an integrated slit lamp and a wide-angle contact lens appeared to be a feasible visualization technique associated with satisfactory anatomical and functional outcomes in this retrospective series. This approach provided adequate visualisation of the peripheral retina to perform SB, and was associated with a high single-surgery success rate and a manageable complication rate. Notably, the entire procedure can be performed using the standard microscope setup, which may simplify the surgical workflow and facilitate ergonomics and teaching. Further prospective studies with larger sample sizes are warranted to confirm these findings.

## Supplementary Information

Below is the link to the electronic supplementary material.

Supplementary Material 1

## Data Availability

No datasets were generated or analysed during the current study.
